# Validation and psychometric properties of the Arabic version of Hamilton Depression Rating Scale 7 items (HAMD-7) among non-clinical and clinical samples of Lebanese adults

**DOI:** 10.1371/journal.pone.0285665

**Published:** 2023-05-18

**Authors:** Sahar Obeid, Vanessa Azzi, Souheil Hallit

**Affiliations:** 1 Social and Education Sciences Department, School of Arts and Sciences, Lebanese American University, Jbeil, Lebanon; 2 School of Medicine and Medical Sciences, Holy Spirit University of Kaslik, Jounieh, Lebanon; 3 Applied Science Research Center, Applied Science Private University, Amman, Jordan; 4 Research Department, Psychiatric Hospital of the Cross, Jal Eddib, Lebanon; King Khalid University, EGYPT

## Abstract

**Background:**

The Hamilton Depression Rating Scale (HDRS or HAMD) is widely used scale for depression assessment. A shortened version of the HDRS, composed of 7 items, was implemented. The latter is timesaving compared to the original version, while still providing similar precision. Our objective in this study was to assess the psychometric properties of the Arabic HAMD-7 scale among non-clinical and clinical samples of Lebanese adults.

**Methods:**

In study 1, 443 Lebanese citizens enrolled in this cross-sectional study (June-September 2021). The total sample in study 1 was divided into two subsamples to conduct the exploratory-to-confirmatory factor analysis (EFA-to-CFA). Another cross-sectional study was conducted in September 2022 on another sample of Lebanese patients (independent from the sample of study 1) and included 150 patients attending two psychology clinics. The Montgomery–Asberg Depression Rating Scale (MADRS), Lebanese Depression Scale (LDS), Hamilton Anxiety Scale (HAM-A) and Lebanese Anxiety Scale (LAS) were used to assess the validity of the HAMD-7 scale.

**Results:**

The results of the EFA (subsample 1; study 1) showed that the HAM-D-7 items converged into a one-factor solution (McDonald’s ω = .78). The CFA (subsample 2; study 1) confirmed the one-factor solution obtained in the EFA (ω = .79). CFA indicated that fit of the one-factor model of the HAM-D-7 was acceptable: χ^2^/df = 27.88/14 = 1.99, RMSEA = .066 (90% CI = .028, .102), SRMR = .043, CFI = .960, TLI = .939. All indices suggested that configural, metric, and scalar invariance was supported across gender. The HAMD-7 scale score positively correlated with the MADRS (r = 0.809; p<0.001), LDS (r = 0.872; p<0.001), HAM-A (r = 0.645; p<0.001) and LAS (r = 0.651; p<0.001) scales scores. The optimal cutoff score between healthy individuals and depressive patients for the HAMD-7 was at a value = 5.50 (Se = 82.8% and Sp = 62.4%). The positive and negative predicted values for the HAMD-7 were 25.1% and 96.0%, respectively. The positive and negative likelihood ratios were 2.20 and 0.28 respectively. No significant difference was found between the non-clinical total sample (study 1) and the clinical sample (study 2) in terms of HAM-D-7 scores (5.24 ± 4.43 vs 4.54 ± 5.06; t(589) = 1.609; p = .108).

**Conclusion:**

Psychometric properties of the Arabic HAMD-7 scale are satisfactory, warranting its use clinically and in research. This scale seems highly efficient for ruling out depression; however, those with positive scores need a referral to a mental health professional for deeper evaluation. HAMD-7 might be self-administered by non-clinical subjects. Future studies are recommended to additionally confirm our results.

## Background

Depression is an emotional disorder causing dysfunction in the capacities and functions of daily life. While considered a public health concern, depression is classified among the disorders that ponder most heavily on individuals, families, and society [1]. Depression, as a mental disorder, portrays the presence of anhedonia (loss of pleasure), physical fatigue with reduced energy (anergy), a feeling of culpability, lack of self-assurance, disrupted sleep and appetite, with cognitive disorders, in particular, an attenuation of concentration and memory problems [2]. Likewise, depression often begins in early life, recurring at different stages, hence correlated with various negative consequences, encompassing higher morbidity and mortality rates and reduced quality of life [3]. Consequently, depression is the foremost source of incapacity globally regarding the number of disoriented years attributable to infirmity [4].

Previously conducted studies revealed a prevalence of depression of 4.4% before the COVID-19 pandemic [5], while this number increased by 27.6% due to that pandemic [6]. In Lebanon, a recent study showed even higher rates (59.7%) [7]. Across all psychiatric disorders, depression and anxiety usually co-occur [8]. A worldwide study stated that 45.7% of subjects with lifetime major depressive disorder had at least one anxiety disorder episode [9]; in fact, one or more anxiety disorders were present in 41.6% of people with 12-month severe depression.

The assessment of depressive symptoms depends on the use of standardized tools, given the lack of clinically relevant biomarkers for depression. Self-report scales, a rapid and reliable choice in the initial assessment of depression, are widely applied clinically and in the research field. These scales include but are not limited to the Beck Depression Inventory (BDI) [10], Montgomery-Asberg Depression Rating Scale (MADRS) [11], Lebanese Depression Scale (LDS-19) [12], Patient Health Questionnaire-9 (PHQ-9) [13], Center for Epidemiological Studies Depression Scale (CES-D) [14] and the Zung Self-Report Depression Scale [15].

The Hamilton Depression Rating Scale (HDRS or HAMD), published in 1960 [16], is the furthermost commonly used scale to assess depression [17]. The original scale comprises 21 items, with the last four (diurnal variation, depersonalization/derealization, paranoid symptoms, and obsessional and compulsive symptoms) deliver additional clinical data while not affecting the final score. The other items are graded on a range of 3–5 points, and the overall score determines how severe the patient’s depression is. Since its publication, it has evolved into the benchmark for diagnosing depression [18], however, the HAMD is not a diagnostic instrument [19]. The HAMD-17 has been validated in different languages, including but not limited to French [20], Turkish [21], Arabic [22] and Chinese [23]. The French version consists of three factors, the Arabic one four factors, the Chinese version five factors and the Turkish one six factors. Methodological limitations, cultural context and clinical groups studied [24] might explain the differences in the results at the factor analysis level. Approaches within the item response theory framework, which are especially pertinent to the field of transcultural psychiatry, allow for a thorough study of cultural variations in the responses to individual items [25].

A shortened version of the HDRS composed of 7 items was implemented in 2003 to assess the depression gravity according to the fourth version of the Diagnostic and Statistical Manual of Mental Disorders criteria [26]. An extraction technique was used to identify the most commonly endorsed and sensitive depressive items from the HAMD-17, a standardized clinician-rated depression scale [26]. It is interesting to note that this shortened version aimed to identify a cutoff score and evaluate the effectiveness of the current treatment by differentiating between symptomatic remission and full clinical response to treatment. Results of the latter study showed that a score of ≤3 indicate a complete remission of symptoms with the HDRS [26]. Noting that the HAMD-7 scale provides the same level of accuracy while being timesaving than the original version [27].

A single published review regarding the validity of the HAMD scale [18] criticized its factorial and content validity, while its convergent, discriminant, and predictive validity are generally satisfying. This was supported by previous authors [28] who showed a high correlation between the HAMD-17 and BDI scores (r = 0.73). The only subscale that has been repeatedly evaluated and recommended as a formal measure in itself is the 6-item melancholia subscale [29]. The latter reported the validity of the HAMD-6 for the assessment of the central symptoms of depression [30]. In 1993, Gibbons et al. [31] recommended a more condensed, one-dimensional tool composed of 8 items (HAMD-8) for the assessment of depression severity.

Only a few cross-cultural studies are included in Bagby and colleagues’ review [18]. Therefore, it is unclear whether the review’s conclusions apply to non-Western populations. In this context, a systematic review conducted in 2019 revealed that a number of depression items remain common in different cultures, however, other items differ between cultures as seen by the variability of Cronbach’s alpha values and number of factors retained [32].

The HAMD-7 scale was previously validated in Arabic in Saudi Arabia [27] and demonstrated to have good test-retest reliability [27]; however, there is a need to implement research among other diagnosed Lebanese patients with depression in view of assessing its adequacy in evaluating the intensity of depressive symptoms, hence the importance of our study. Furthermore, Lebanon differs from Saudi Arabia on cultural, economic, and political levels. For example, in Lebanon the economy is smaller than the average Arab country, do not derive a significant share of his revenues from natural resources, has a significantly more than average quality of democracy/participatory political system in opposite to Saudi Arabia classified as "politically restrictive with high gender inequality" [33]. Therefore, our main objective in this study was to validate the HAMD-7 scale among non-clinical and clinical samples of Lebanese adults, and compare its psychometric properties with two accepted measurement tools, MADRS and LDS-19. We hypothesize that the items of HAMD-7 would load on a one-factor solution.

## Methods

### Ethics approval and consent to participate

The Psychiatric Hospital of the Cross Ethics and Research Committee agreed on this study protocol (HPC-023-2021). Submitting the form online was considered equivalent to obtaining a written informed consent.

### Study 1 (non-clinical/community sample)

#### General design

This cross-sectional study was performed between June and September 2021, during which 443 Lebanese citizens were recruited from the community from all governorates via a chain sampling snowball technique and filled an online survey. The research team asked acquaintances to forward the link along to their friends and family members. The link was distributed to participants via social media applications (WhatsApp, Instagram, Messenger, etc.). Everyone above 18 years old was qualified for the study. Mundfrom et al. suggest a minimum sample based on 3–20 times the number of items of the scale [34]. Therefore, since the short form of the HAM-D scale is composed of 7 items, two sample of 140 participants each were needed at least to perform the exploratory and confirmatory factor analyses respectively.

### Participants characteristics (sample 1)

The mean age of the total sample was 31.71 ± 13.90 years, with 57.1% females; 193 (43.6%) had depressive symptoms (scores ≥4). A significantly higher percentage of participants who had a physician-diagnosis with depression had a secondary level of education or less, whereas a higher mean age was significantly found in patients who had a physician-diagnosis with depression (Table 1).

**Table 1:** Sociodemographic characteristics of the sample population.

	Total	Physician-diagnosed with depression		X^2^ or t	df	*p*
	(N = 443)	No (N = 381)	Yes (N = 58)			
**Sex**				0.274	1	0.601
Male	190 (42.9%)	165 (87.8%)	23 (12.2%)			
Female	253 (57.1%)	216 (86.1%)	35 (13.9%)			
**Marital status**				2.121	1	0.145
Single / widowed / divorced	261 (58.9%)	229 (88.8%)	29 (11.2%)			
Married	182 (41.1%)	152 (84.0%)	29 (16.0%)			
**Education level**				21.082	1	**<0.001**
Secondary or less	165 (17.0%)	108 (76.1%)	34 (23.9%)			
University	663 (62.1%)	273 (91.9%)	24 (8.1%)			
**Monthly income**				0.561	3	0.905
No income	90 (20.3%)	75 (86.2%)	12 (13.8%)			
Less than 1000 $	162 (36.6%)	138 (85.7%)	23 (14.3%)			
Between 1000–2000 $	107 (24.2%)	95 (88.8%)	12 (11.2%)			
Higher than 2000 $	84 (19.0%)	73 (86.9%)	11 (13.1%)			
	**Mean ± SD**					
**Age (in years)**	31.71 ± 13.90	31.24 ± 13.80	35.21 ± 14.39	2.030	437	**0.043**

Numbers in bold indicate significant *p* values.

### Study 2 (clinical sample)

We conducted another cross-sectional study in September 2022 on another sample of Lebanese patients (independent from the sample enrolled in study 1); 150 patients, attending two psychology clinics, enrolled in study 2 (mean age = 33.21 ± 14.26 years; 52% females; 29.3% married and 64.7% university level of education). The HAM-D-7 scale’s items were administered via a face-to-face interview with the clinical psychologist.

### Measurement

The Arabic questionnaire consisted of questions about sociodemographic aspects (sex, age, marital status, educational level, and monthly income). It also asked participants to self-report a physician diagnosis of depression and anxiety (no/yes type of answer). It included the following scales:

#### Hamilton Depression Rating Scale (HAMD-7)

The HAMD was used to measure depression that occurred in the last 7 days [35]. It consists of 7 items (e.g. “depressed mood: Have you been feeling down or depressed this past week?—How often have you felt this way, and for how long?”), rated on a 5-point scale from 0 (not present) to 4 (severe). Higher scores indicate higher depression. Scores ≥4 indicate the presence of depressive symptoms [27].

#### Montgomery–Asberg Depression Rating Scale

The MADRS is validated in Lebanon [36]. It is composed of 10 items assessing the severity of depression exhibited over the preceding two weeks [11] (e.g. “Apparent sadness”, “Reported sadness”). Items are scored from 0 to 6 [37].

#### Lebanese Depression Scale

The LDS‐19 scale evaluates depression over the last 2 weeks and is composed of 19 items (e.g. "Retardation (slowness of thought and speech, impaired ability to concentrate, decreased motor activity”)) [38]. Higher scores indicate higher depression.

#### Hamilton Anxiety Scale

The Hamilton Anxiety Scale (HAM-A) is validated in Lebanon [39] and includes 14 items [40] rated from 0 (symptoms not present) to 4 (very extreme symptoms). The higher the scores, the more the anxiety (e.g. “Anxious mood: Worries, anticipation of the worst, fearful anticipation, irritability”).

#### Lebanese Anxiety Scale

It contains 10 items and is validated among adults and adolescents [41,42] for the assessment of anxiety symptoms experienced during the past week (e.g. “I have insomnia (Difficulty in falling asleep, broken sleep, unsatisfying sleep and fatigue on waking, dreams, nightmares, night terrors)”. Higher scores indicate higher levels of anxiety.

### Statistical analysis

SPSS software version 23 was used to perform the statistical analysis. Missing values represented less than 10% of the total and were replaced with the mean value [43]. The HAMD-7 score’s normal distribution was validated by its skewness (= 0.722) and kurtosis (= .224) values varying between ±1 [44]. The Chi-square test was used to compare two percentages. Student t test was used to compare two means. *P* <0.05 was envisaged as significant.

We employed an exploratory-to-confirmatory (EFA-to-CFA) factor analysis method to investigate the HAMD-7 factor structure [45]. We divided Sample 1 using the SPSS-generated random approach to provide suitable sample sizes for both EFA and CFA. There were no significant differences between the two subsamples in terms of mean age, *t*(824) = 1.52, *p* = .129, *d* = .11, and BMI, *t*(824) = 0.07, *p* = .944, *d* = .004, as well as the distribution of women and men, χ^2^(1) = .015, *p* = .901, of single and married participants, χ^2^(1) = .083, *p* = .773, education level χ^2^(1) = 1.603, *p* = .205, and monthly income, χ^2^(3) = 3.936, *p* = .268.

First, we computed a robust EFA using the principal analysis component with the first split-half subsample using the FACTOR software [46]. We confirmed that all conditions related to item-communality [47], average item correlations, and item-total correlations [48], Weighted Root Mean Square Residual (WRMR) [49], Kaiser-Meyer-Olkin (KMO) measure and Bartlett’s test of sphericity [50], were met. The dimensionality of the instrument was determined through the optimal implementation of Parallel Analysis [51], using the Pearson correlation matrix. Items retained when having a factor loading >0.4 and communality >0.3 [52].

CFA was performed using SPSS AMOS v.29 using the maximum likelihood estimation methods based on the factor solution obtained in the EFA. Multivariable normality was verified since the Bollen-Stine bootstrap p-value was 0.098 (>0.05). The root mean square error of approximation (RMSEA), Tucker Lewis Index (TLI), and Comparative Fit Index (CFI) assessed the goodness-of-fit of the model [53]. Values of RMSEA ≤0.08, and CFI and TLI ≥0.90 stipulate a good-fitting model [53]. The average variance extracted (AVE) values of ≥ .50 ensured convergent validity [54].

*Gender invariance*. We applied multi-group CFA on the complete sample from study 1 to explore the gender invariance of HAMD-7 scores [55]. Measurement invariance was assessed at the configural, metric, and scalar levels [56], evidenced if ΔCFI ≤ .010 and ΔRMSEA ≤ .015 or ΔSRMR ≤ .010 [55,57].

McDonald’s ω was chosen to estimate reliability due to known issues with the application of Cronbach’s α [58] (values >.70 considered adequate [59]).

The cutoff point of the scale versus physician-diagnosed depression was determined using the Receiver-Operating Characteristics (ROC) curve; sensitivity, specificity, and negative and positive predictive values were calculated for the HAMD-7 scale; for this purpose, we relied on the participant’s self-report of physician diagnosis of depression (yes/no). In addition, positive likelihood ratio was calculated according to the following formula: $LR+=\frac{Sensitivity}{1-Specificity}$, whereas negative likelihood ratio was calculated according to: $LR-=\frac{1-Sensitivity}{Specificity}$.

Finally, to evaluate convergent validity, we tested the correlations between HAMD-7 and MADRS, LDS, HAM-A and LAS scales since depression and anxiety usually coexist [60].

## Results

McDonald’s omega values in the total sample (study 1) were as follows: MADRS (ω = 0.85), LDS (ω = 0.87), HAM-A (ω = 0.95) and LAS (ω = 0.94). The mean HAM-D score in sample 1 was 5.24 ± 4.43 and 4.54 ± 5.06 in sample 2.

### Principal Component Analysis (subsample 1; study 1)

In both men and women, the advised number of dimensions according to the parallel analysis was one. The common variance explained was 49.15% and 40.54% respectively. The WRMR values were also adequate (.131; 95% CI .114-.142 and .120; 95% CI .106-.130) respectively. The factor loadings are shown in Table 2. McDonald’s ω was satisfactory in this subsample (ω = .78), in men (ω = .82) and in women (ω = .76).

**Table 2:** Items of the HAM-D-7 in English and factor loadings derived from the Exploratory Factor Analyses (EFA) in the first split-half subsample (study 1) in men and women, and standardised estimates of factor loadings from the Confirmatory Factor Analysis (CFA) in the second split-half subsample (study 1).

Item	EFA		CFA
	Men	Women	
1. Depressed mood	.59	.67	.59
2. Feelings of guilt	.74	.72	.54
3. Suicide	.68	.55	.45
4. Work and activities	.69	.62	.53
5. Anxiety psychic	.72	.70	.63
6. Anxiety somatic	.79	.63	.74
7. General somatic symptoms	.68	.55	.62

### Confirmatory Factor Analysis (subsample 2; study 1)

Fit indices for the CFA of the one-dimensional model of the HAM-D-7 was acceptable: χ^2^/df = 27.88/14 = 1.99, RMSEA = .066 (90% CI = .028, .102), SRMR = .043, CFI = .960, TLI = .939. The standardised estimates of factor loadings (Table 2) and AVE = .55 were acceptable. McDonald’s omega was good in the second subsample (ω = .79).

### Gender invariance

All indices suggested that configural, metric, and scalar invariance was supported across gender (Table 3). The Student t test results showed that a higher mean HAMD-7 score was seen in women (*M* = 5.88, *SD* = 4.39) compared to men (*M* = 4.52, *SD* = 4.55) in the second subsample from study 1, *t*(211) = 2.201, *p* = .029, *d* = .304.

**Table 3:** Measurement invariance across gender in the total sample from study 1.

Model	χ^2^	*df*	CFI	RMSEA	SRMR	Model Comparison	Δχ^2^	ΔCFI	ΔRMSEA	ΔSRMR	Δ*df*	*p*
Configural	51.49	28	.928	.063	.062							
Metric	62.53	34	.913	.063	.070	Configural vs metric	11.04	.015	< .001	.008	6	.087
Scalar	75.24	41	.895	.063	.072	Metric vs scalar	12.71	.018	< .001	.002	7	.079

*Note*. CFI = Comparative fit index; RMSEA = Steiger-Lind root mean square error of approximation; SRMR = Standardised root mean square residual.

### Convergent validity (total sample)

The HAMD-7 scale score positively correlated with the MADRS (r = 0.809; p<0.001), LDS (r = 0.872; p<0.001), HAM-A (r = 0.645; p<0.001) and LAS (r = 0.651; p<0.001) scales scores.

### Validity measures (ROC curve) (sample 1)

**Fig 1:**
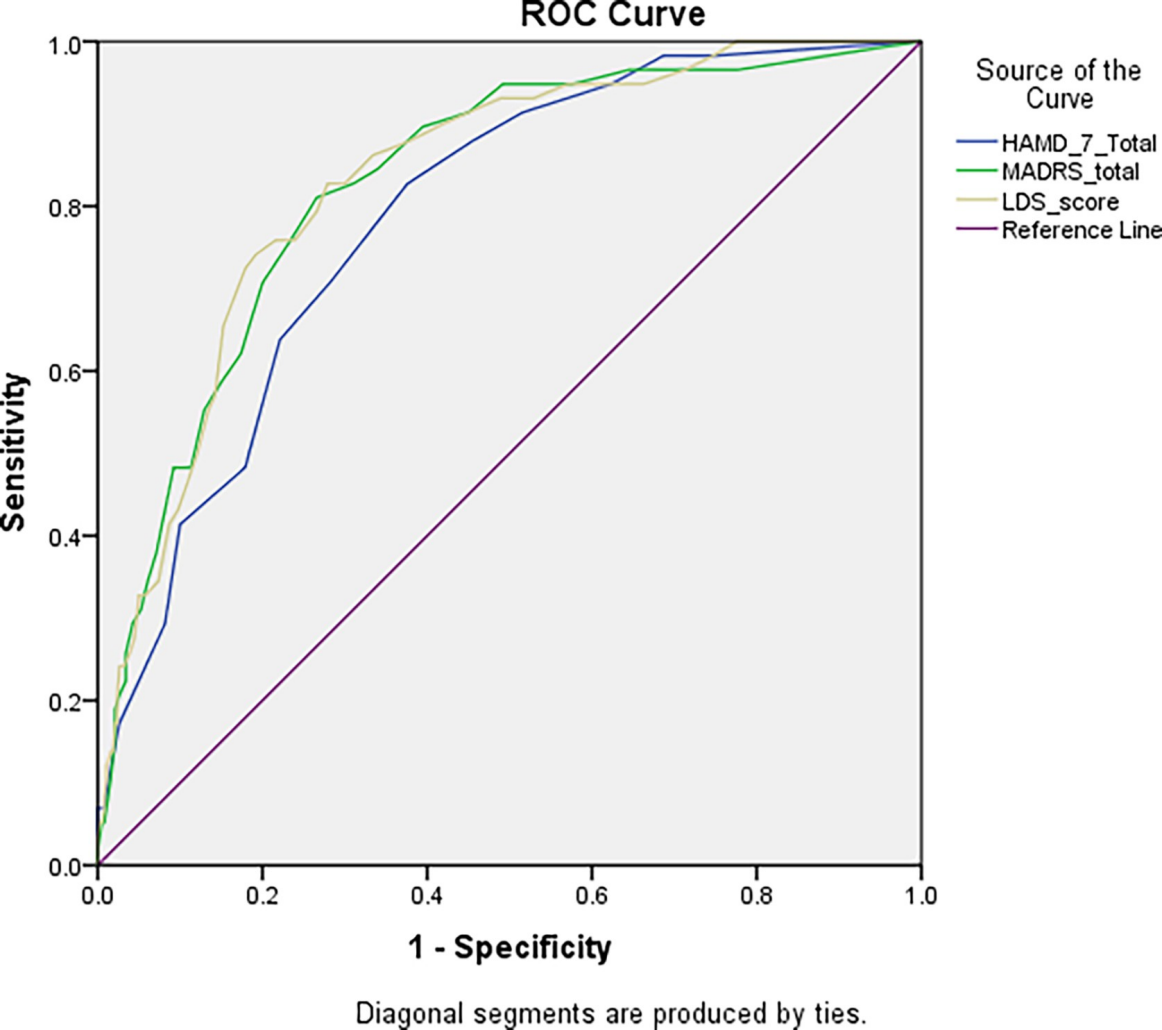
ROC curve of the depression scales. Patients with a self-report physician’s diagnosis of depression were analyzed (sample 1). Area under the curve of LDS-19 scale = 0.833 [0.781–0.885] (p<0.001); at value = 16.50, Se = 82.8% and Sp = 72.1%. Area under the curve of HAMD-7 scale = 0.788 [0.731–0.844] (p<0.001); at value = 5.50, Se = 82.8% and Sp = 62.4%. Area under the curve of MADRS scale = 0.827 [0.773–0.882] (p<0.001); at value = 9.50, Se = 81.0% and Sp = 73.4%.

The performance of the HAM-D-7 scale scores in terms of classifying participants by their depression status (yes/no; self-report physician diagnosis of depression) was evaluated with ROC curve analysis The HAMD-7 scale had a smaller area under the curve compared to the LDS and MADRS scales. The HAMD-7’s ideal cutoff score between healthy people and depressive patients was at a value of 5.50 (Se = 82.8% and Sp = 62.4%) (Fig 1). This indicates that this cutoff value, the HAMD-7 scale is capable of capturing persons with depressive symptoms by 82.8%, whereas it is capable to capturing persons who are not depressed by 62.4%.

### Predictive values (total sample)

The positive and negative predicted values for the HAMD-7 were 25.1% and 96.0%, respectively.

### Likelihood ratios (total sample)

The positive and negative likelihood ratios were 2.20 and 0.28 respectively.

### Comparison of the HAM-D scores between the clinical and non-clinical samples

No significant difference was found between the non-clinical total sample (study 1) and the clinical sample (study 2) in terms of HAM-D-7 scores (5.24 ± 4.43 vs 4.54 ± 5.06; t(589) = 1.609; p = .108).

## Discussion

### Factorial validity supporting the one-factor model

We examined the psychometric properties of the HAMD-7 among non-clinical and clinical samples of Lebanese adults. Researchers are constantly criticized for the administration of long surveys, with other scales created to screen for the presence of depressive symptoms (such as the HAMD-17 and the LDS) being lengthy and time-consuming. Therefore, we found a necessity to evaluate the psychometric properties of the HAMD-7 scale. Our results confirm the psychometric properties of the one-factor solution. Furthermore, this scale can be completed in a couple of minutes.

### Internal consistency and convergent validity

The HAM-D 7 items appeared to be internally consistent, reaching a value of .78 and .79 respectively, higher than the value obtained in the previous Arabic validation in Saudi Arabia (Cronbach’s alpha values < .75 in the pre-and post-tests). Concerning the convergent validity of the proposed factorial solution, the HAMD-7 total score correlated exceptionally well with other validated depressive symptoms’ screening scales (LDS and MADRS). It also highly correlated with anxiety scales (HAM-A and LAS), proving the divergent validity of the scale. Summing up, the current study’s findings support the construct validity of the Arabic HAMD-7. In addition, the non-significant difference between the clinical and non-clinical samples suggest that there is no difference in the administration of the scale between a healthcare professional and the participant him/herself (self-adminsitered). Participants may now have more privacy when answering the questions, be at ease and more willing to provide honest answers.

### ROC curve

The area under the curve of the HAMD-7 was lower than that of the LDS and MADRS scales. This was kind of expected since the latter scales include items that cannot be found in the HAMD-7. Despite the aforementioned benefits of the HAMD-7, there are some drawbacks to this brief version. For example, insomnia, disturbed appetite, loss of libido, and hypochondriasis are only a few of the HAMD-17 items that the HAMD-7 does not include, so it may not detect some symptoms that the HAMD-17 may detect [61]. However, a high correlation with the MADRS and Clinical Global Impression Scale, as well as acceptable levels of sensitivity and specificity, indicate that the HAMD-7’s briefness does not seem to to jeopardize crucial details on patient progress and outcome [35].

The scale has high sensitivity and moderate specificity, indicating that participants scoring less than 5.5 can be considered not suffering from depression. Furthermore, the NPV value was excellent (96%), which means that the HAMD-7 scale can be used to rule out depression, whereas the PPV was low (25.1%), reflecting that this scale cannot be used for the diagnosis/screening of depressive symptoms. Those findings are similar to those obtained with the MADRS [62] and LDS [12] scales. Hence, a practitioner’s diagnosis is crucial to the screening test’s validation.

### Clinical implications

Results of our study suggest that the HAMD-7, Arabic version, may be a possibly valuable clinical tool for clinicians and researchers aiming to assess depression severity among Arab subjects. Such research from an understudied cultural setting, on a subject that is culturally dependent, could support the instrument’s external validity [63]. Furthermore, we recommend clinical psychologists and educators to use the shortened version of the HAMD-7 to conduct a thorough assessment into the presence of depression among subjects who are more likely to experience depressive symptoms and to take a holistic approach to manage their condition. Additionally, young adults and those who have had significant life events, chronic medical conditions, or mental health disorders should regularly be screened for depression with the necessary management following.

### Limitations

These results cannot be attributed to the entire population since the sample had a relatively low mean age, with the majority having a university education level. In addition, patients tend to exaggerate the severity of their symptoms. The snowball technique followed in the data collection, the inability to know the refusal rate/response rate implicate a selection bias because of the respondent-driven sampling. We failed to test the criteria validity that needs clinical diagnosis according to the standard clinical test (physician’s diagnosis). Additionally, relying only on a self-report of being diagnosed with depression (yes or no) might not be a reliable criterion to differentiate clinical and non-clinical populations to establish a cut-off. Other psychometric properties of the scale (test-retest) are recommended to strengthen our results. Divergent validity was tested using scales that screen for anxiety; literature has discussed whether anxiety and depression are two separate constructs or not, therefore, future studies should assess the divergent validity of the HAM-D 7 items using other scales.

### Conclusion

The primary psychometric properties obtained for the Arabic HAMD-7 are satisfactory, warranting this short-scale use clinically and in research. This scale seems highly efficient for ruling out depression; however, those with positive scores need a referral to a healthcare practitioner for deeper evaluation. HAMD-7 might be self-administered by non-clinical subjects. Future studies are recommended to additionally confirm our results.
